# Supplementary material for: Controlled Structure of Polyester/Hydroxyapatite Microparticles Fabricated via Pickering Emulsion Approach
Source: Polymers (Basel). 2022 Oct 13;14(20):4309. doi: 10.3390/polym14204309 (PMC9609128; doi:10.3390/polym14204309)
Supplement: Supplementary file 1 [file polymers-14-04309-s001.zip › polymers-1935446-supplementary.pdf]

polymers

Supplementary materials: Controlled Structure of Polyester/Hydroxyapatite Microparticles Fabricated via Pickering Emulsion Approach

Nikita V. Minaev, Svetlana A. Minaeva, Anastasia A. Sherstneva, Tatiana V. Chernenok, Yulia K. Sedova, Ekaterina D. Minaeva, Vladimir I. Yusupov, Tatiana A. Akopova, Peter S. Timashev and Tatiana S. Demina

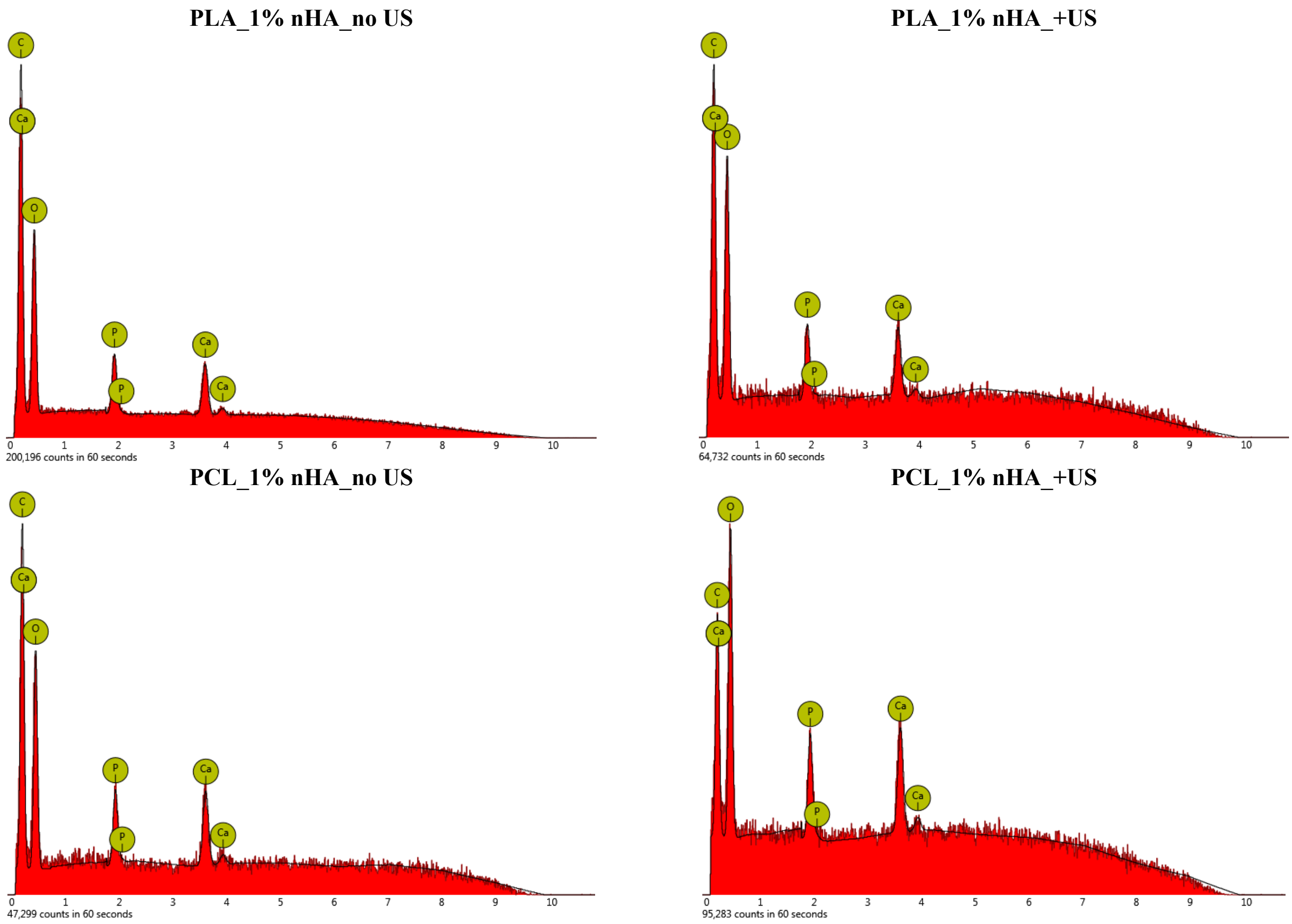

PCL_1% nHA_no US (EDX data at various surface points)

Point 1: C: 14.6±0.2; O: 72.2±0.2; Ca: 8.2±0.5; P: 5.0±0.2

Point 2: C: 9.3±0.3; O: 73.6±0.2; Ca: 14.8±0.5; P: 2.3±1.2

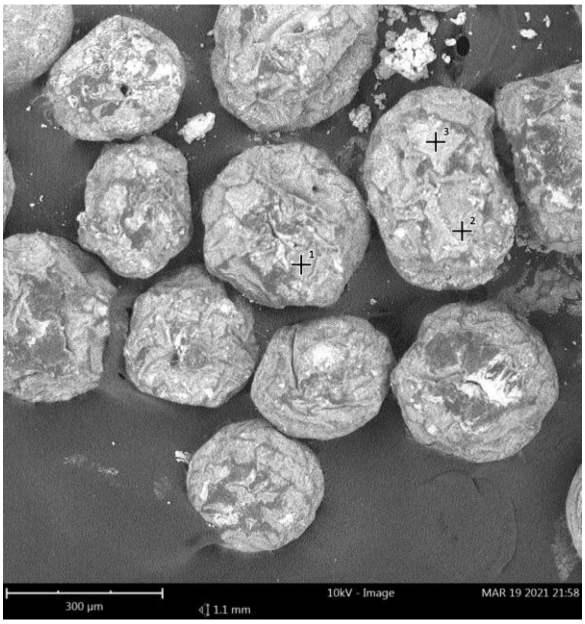

Point 3: C: 7.8±0.3; O: 67.5±0.3; Ca: 19.9±0.6; P: 4.8±0.8

Figure S1. EDX spectra taken at surface points of the microparticles based on PLA or PCL made using 1 wt% nHA in the aqueous phase and with or without preliminary US treatment.

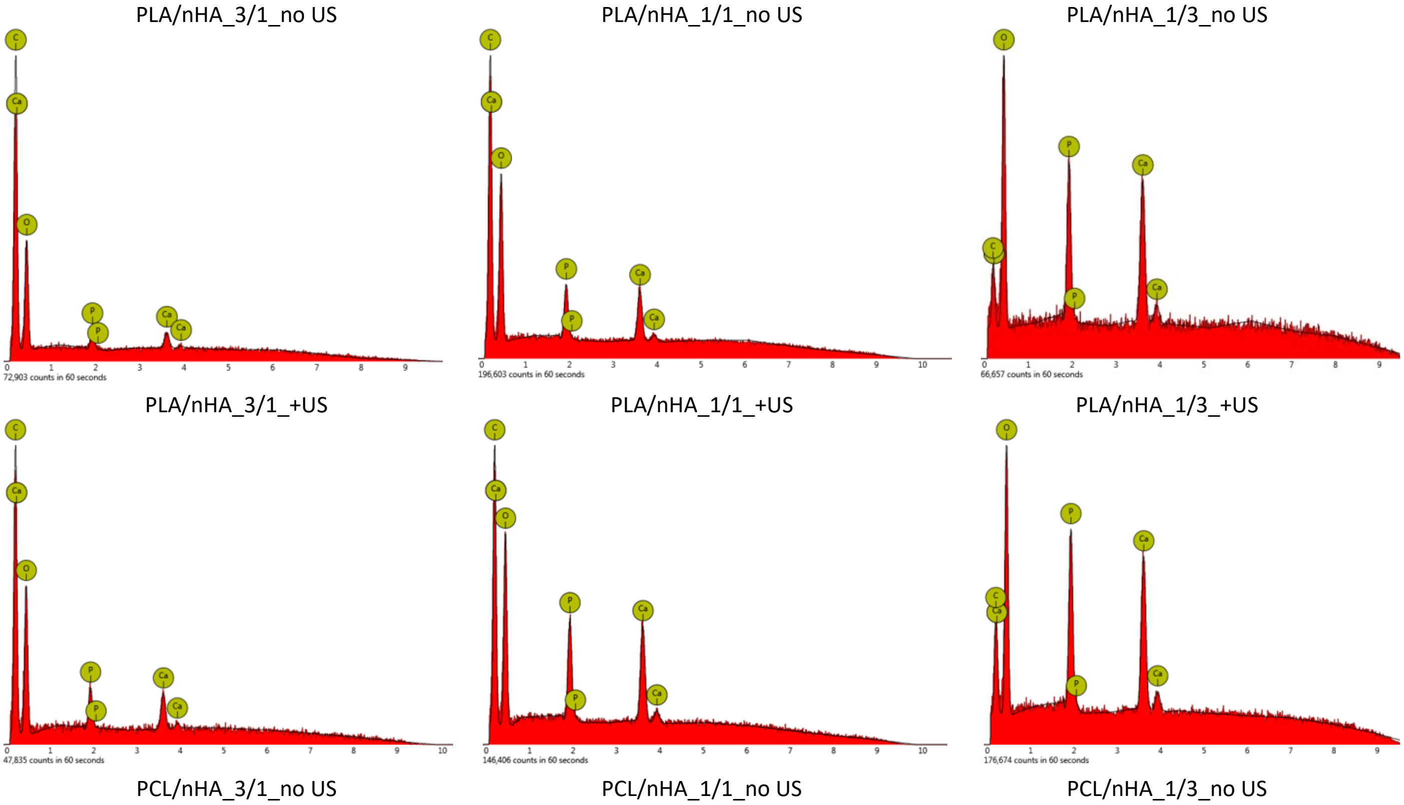

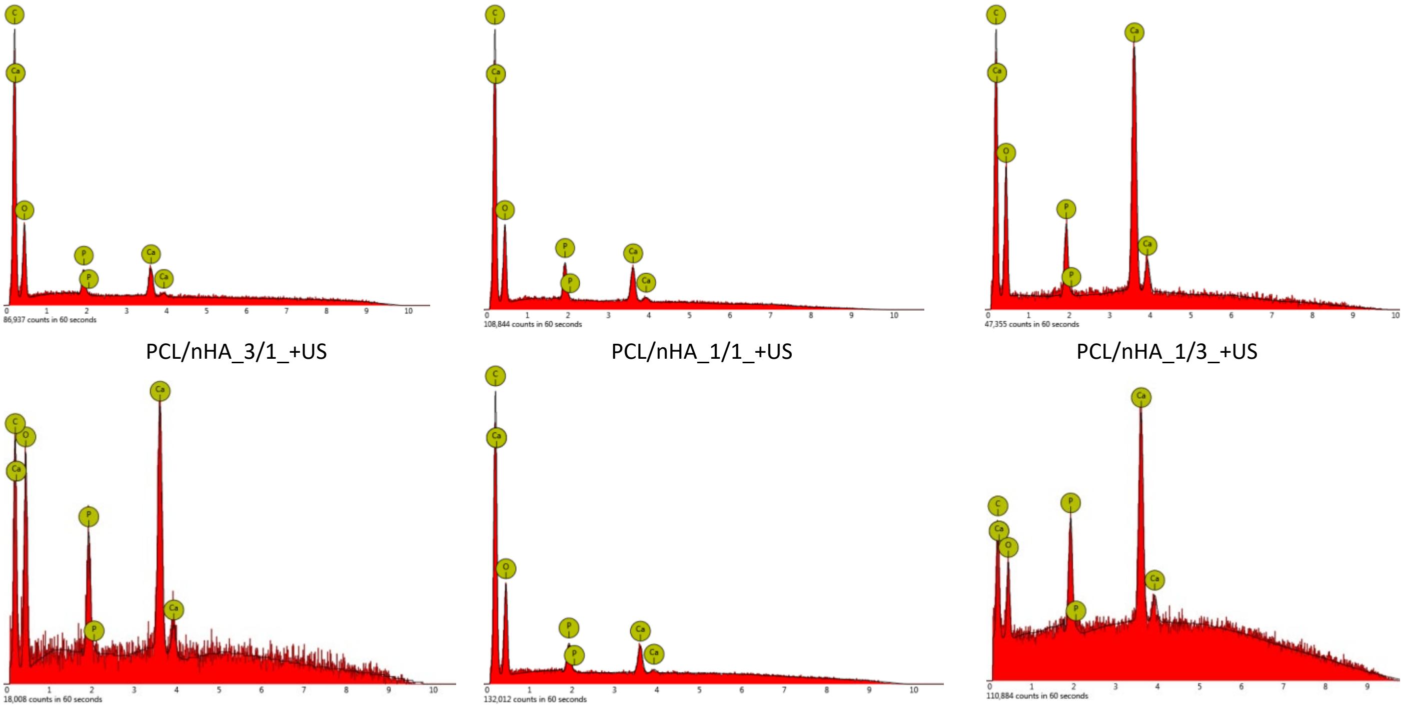

Figure S2. EDX spectra taken at surface points of the microparticles based on PLA or PCL made using various polymer/nHA ratios in the oil phase and with or without preliminary US treatment of oil phase.

Table S1. Mean atomic concentrations at the surface of the hybrid microparticles as a function of oil phase composition and the US pre-treatment.

**Sample**		**Atomic concentration, at.%**							
		**no US**				**+US**			
		**C**	**O**	**Ca**	**P**	**C**	**O**	**Ca**	**P**
**PLA/nHA**	**3/1**	23.3□0.3	69.7□0.2	3.9□0.5	3.2□0.5	18.3□0.1	71.2□0.2	6.2□0.3	4.3□0.4
	**1/1**	11.0□0.1	64.1□0.2	16.6□0.2	8.4□0.2	8.5□0.1	58.6□0.1	21.9□0.3	11.0□0.2
	**1/3**	2.8□0.8	76.8□0.1	12.1□0.3	8.3□0.2	2.5□0.5	59.0□0.1	24.8□0.2	13.6□0.1
**PCL/nHA**	**3/1**	17.7□0.2	58.2□0.1	17.0□0.3	7.2□0.5	7.4□0.2	68.1□0.3	18.8□0.3	5.7□0.7
	**1/1**	16.8□0.2	56.0□0.1	17.8□0.3	9.4□0.3	16.8□0.2	61.0□0.2	14.7□0.3	7.5□0.3
	**1/3**	5.3□0.2	44.7□0.2	43.8□0.3	6.2□0.2	3.9□0.6	36.3□0.3	47.6□0.3	12.2□0.4

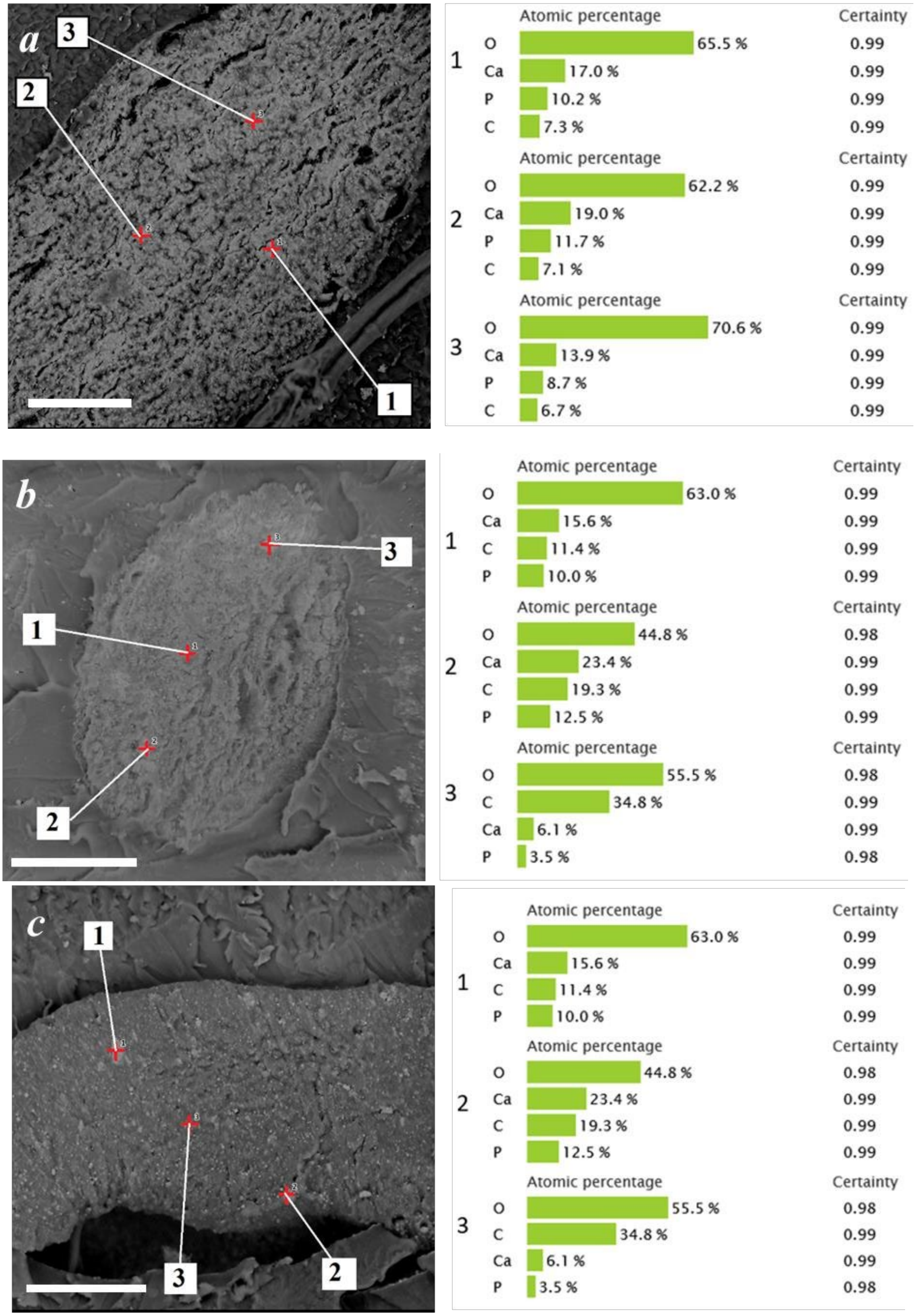
a
3
2
1
1
Atomic percentage
Certainty
O
65.5 %
0.99
Ca
17.0 %
0.99
P
10.2 %
0.99
C
7.3 %
0.99
2
Atomic percentage
Certainty
O
62.2 %
0.99
Ca
19.0 %
0.99
P
11.7 %
0.99
C
7.1 %
0.99
3
Atomic percentage
Certainty
O
70.6 %
0.99
Ca
13.9 %
0.99
P
8.7 %
0.99
C
6.7 %
0.99
b
3
1
2
1
Atomic percentage
Certainty
O
63.0 %
0.99
Ca
15.6 %
0.99
C
11.4 %
0.99
P
10.0 %
0.99
2
Atomic percentage
Certainty
O
44.8 %
0.98
Ca
23.4 %
0.99
C
19.3 %
0.99
P
12.5 %
0.99
3
Atomic percentage
Certainty
O
55.5 %
0.98
C
34.8 %
0.99
Ca
6.1 %
0.99
P
3.5 %
0.98
c
1
3
2
1
Atomic percentage
Certainty
O
63.0 %
0.99
Ca
15.6 %
0.99
C
11.4 %
0.99
P
10.0 %
0.99
2
Atomic percentage
Certainty
O
44.8 %
0.98
Ca
23.4 %
0.99
C
19.3 %
0.99
P
12.5 %
0.99
3
Atomic percentage
Certainty
O
55.5 %
0.98
C
34.8 %
0.99
Ca
6.1 %
0.99
P
3.5 %
0.98

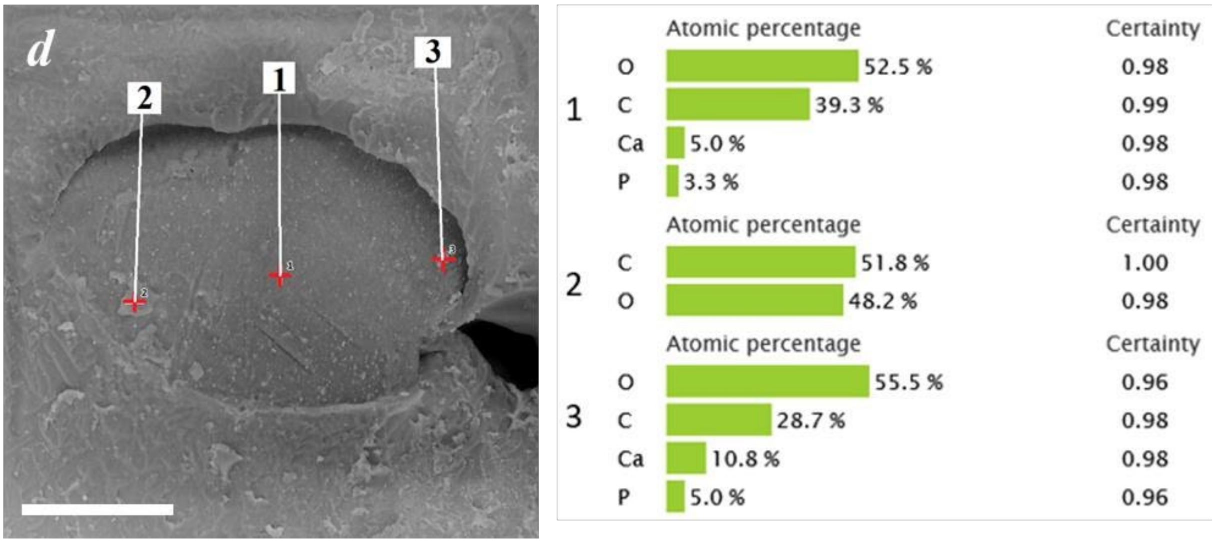

Figure S3. SEM images and EDX data of probes taken at various points of the cross-sections of the microparticles based on PLA (a,b) or PCL (c,d) using at 1/1 polymer/nHA ratio and made with (b,d) or without (a,c) preliminary US treatment of oil phase Scale bars: 50 μm.
